# Isolated Facet Joint Arthritis in Juvenile Idiopathic Arthritis

**DOI:** 10.7759/cureus.12596

**Published:** 2021-01-09

**Authors:** Giulia Martone, Richard D Thomas, Joseph Keuchle, Rabheh Abdul-Aziz

**Affiliations:** 1 Department of Pediatrics, University at Buffalo Jacobs School of Medicine & Biomedical Sciences, Buffalo, USA; 2 Department of Radiology, John R. Oishei Children's Hospital, Buffalo, USA; 3 Department of Orthopaedics, University at Buffalo Jacobs School of Medicine & Biomedical Sciences, Buffalo, USA; 4 Department of Pediatric Rheumatology, University at Buffalo Jacobs School of Medicine & Biomedical Sciences, Buffalo, USA

**Keywords:** facet joint, zygapophyseal joint, juvenile idiopathic arthritis, child

## Abstract

Juvenile idiopathic arthritis (JIA) is a group of childhood inflammatory arthropathies which has variable clinical presentations and can affect multiple joints including the spine. Arthritis in facet joints is rare and very unusual to be the only presentation of JIA. We report a 16-year-old female who presented with back pain and stiffness, in which CT of the lumbar spine showed evidence of facet joint erosion and pelvis MRI showed facet joint arthritis consistent with juvenile idiopathic arthritis.

## Introduction

Juvenile idiopathic arthritis (JIA) is the most common systemic autoimmune disease of connective tissue affecting children [1]. It is classified into subtypes depending on joints affected, symptoms, and antibody presence, with a wide range of severity [2]. The joints that are most commonly affected by JIA are knees, hands and wrists, hips, and ankles [3]. The majority of JIA cases that affect the spine are located in the cervical region such as synovitis, atlantoaxial subluxation, or ankylosis [4]. Less commonly, ankyloses have been reported of JIA affecting the lumbosacral spine [5], which occurs more predominantly in adolescents [6]. Ankylosis that affects the lumbar and sacral spine is seen in enthesitis-related JIA which affects about 10 to 20% of children with JIA [7].

Prolonged inflammation in the facet joints has been reported predominantly in ankylosing spondylitis and has been found to be distributed evenly amongst all spine levels [8,9]. As a result of the chronic inflammatory state, children are at risk of generalized and periarticular bone loss and delayed growth and puberty [10,11]. Spine abnormalities such as vertebral compression fractures can also occur in the thoracic spine however most are influenced by glucocorticoid-induced osteoporosis [6]. In this case study, we present an adolescent with JIA who had an atypical initial presentation of JIA with isolated and severe lumbar spine involvement.

## Case presentation

We present a 16-year-old previously healthy Caucasian female who presented to her primary care physician with a 12-month history of mid-lower back pain that was worse in the morning, after sitting, and after activity. Her pain was reported as 6-8/10 in severity daily, with morning stiffness for 30 minutes. She denied any history of precipitating trauma, and had no history of psoriasis, dactylitis, or nail dystrophy [12]. Review of systems was negative for recent infection, gastrointestinal, urinary, or ocular symptoms (conjunctivitis or uveitis). 

**Figure 1:**
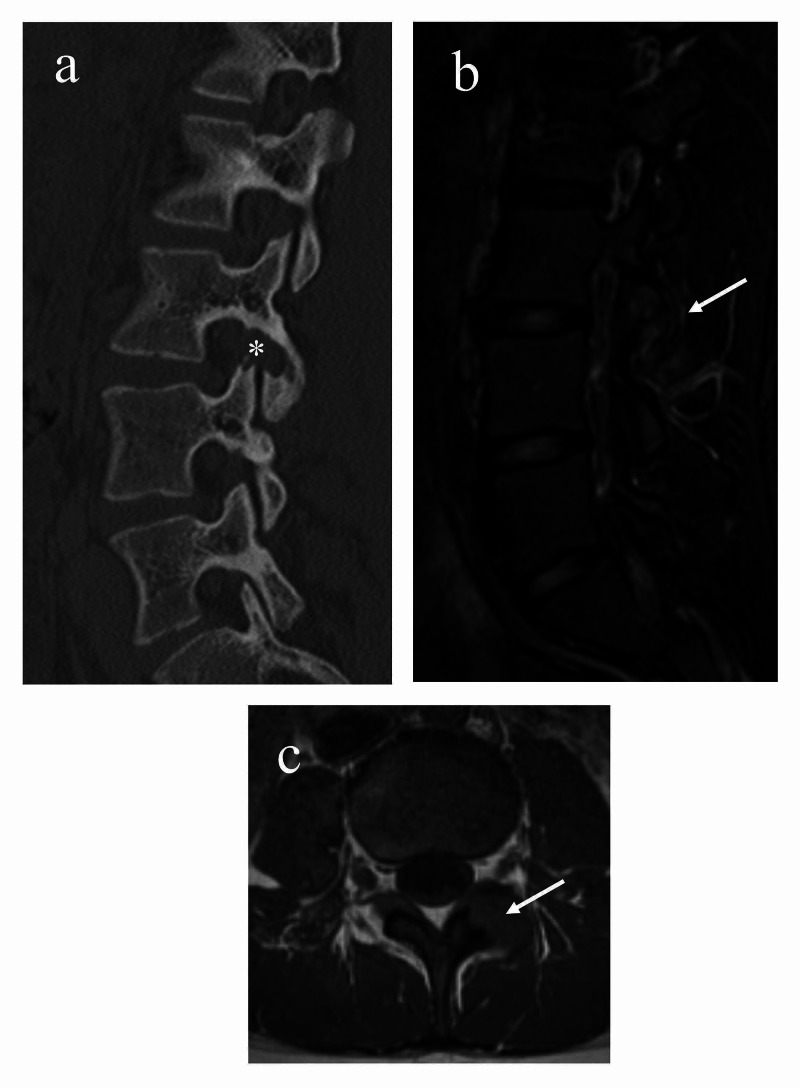
(a) CT Lumbar spine sagittal reformat revealing left L3-L4 facet erosion along both sides of the joint (white asterisk) (b) MRI Lumbar spine T2 sagittal image and (c) T1 axial image showing synovial hypertrophy and osseous erosion at the level L3-L4 facet joint (white arrows).

Spine radiographs requested by orthopedics were normal, however, lumbar Computed Tomography (CT) scan with and without contrast revealed evidence of left facet joint synovial enhancement with erosion along both sides of the joint at level L3-L4 (Figure 1). Physical examination demonstrated pain of left sacroiliac joint with flexion, abduction, and external rotation of the right hip (positive Patrick’s Test), tenderness of lumbar spine, tenderness of left sacroiliac joint, crepitus of both knees, and hypermobility. There was no swelling of any peripheral joints. Initial laboratory findings were negative for anti-nuclear antibody (ANA), rheumatoid factor (RF), anti-citrullinated protein antibody (anti-CCP), and human leukocyte antigen B27 (HLA-B27). Lyme serology was not performed as there was no history of tick bite. Similarly, culture for ureaplasma urealyticum was not performed given the absence of urinary tract symptoms and normal urinalysis. Erythrocyte sedimentation rate (ESR) and C-reactive protein (CRP) were normal at 11mm/hr and 1.02mg/L, respectively. Subsequent contrast-enhanced Magnetic Resonance Imaging (MRI) of the pelvis showed no evidence of sacroiliitis. 

Clinical symptoms, laboratory results, and imaging features yielded a diagnosis of seronegative juvenile idiopathic arthritis [1]. She was started on prednisone 50mg daily for 10 days with clinical improvement of pain from 7/10 to 4/10 after course completion. Naproxen 500mg twice daily provided minimal relief. With persistence of back pain symptoms one month after the initial diagnosis of JIA, she was started on adalimumab 40mg subcutaneous (SQ) every two weeks which was increased to 40mg SQ every week. Back pain improved significantly from 4/10 to 1/10 after one month on this regimen and she was able to resume her baseline activity of softball and hockey six days/week without pain. She was also started on vitamin D 50,000IU weekly and 1g of calcium carbonate daily for eight weeks for vitamin D deficiency (Vitamin D 25 -hydroxy = 21ng/mL) with improvement to a normal level (Vitamin D 25 -hydroxy = 34ng/mL). Repeat imaging was performed six months after adalimumab therapy (40mg SQ every week) with no significant new changes noted.

## Discussion

We report an adolescent girl with a very atypical presentation of JIA. JIA is a heterogeneous group of autoimmune disorders characterized by chronic inflammatory arthritis [1]. The pathogenesis of JIA includes synovial hypertrophy that leads to cartilage formation and damage to subchondral bone [13]. Spinal involvement in JIA can occur rarely, however, it is most frequently found in the cervical region and in patients already diagnosed with JIA [4]. To date, this is the first reported case of facet (zygapophyseal) joint synovitis and erosion in JIA of the lumbar spine as the first presentation of JIA. In 2014, Vendan et al. reported lumbar apophyseal joint synovitis in 38% of cases of adolescents (16.7 years) with enthesitis-related arthritis [14]. A retrospective review on 50 children with severe JIA reported 21% of females had deformed vertebrae, the majority in the lower thoracic spine with no reported facet or apophyseal joint erosion [6]. Facet joint erosion has been reported in an adult patient with gout [15], however, our patient’s normal uric acid level and age makes gout unlikely. It can also be seen in patients with ankylosing spondylitis [8], although the absence of sacroiliitis on MRI lumbar spine suggests this diagnosis is less likely [16]. To date, reactive arthritis has not been reported in the facet joint and the absence of proceeding infection, negative HLA-B27, makes it unlikely [17]. In this unique case, facet joint synovial enhancement and erosion, especially in the lumbar spine is rare for JIA presentation. The disease progression of this adolescent is unknown; however, she reports clinical improvement of symptoms after six months of weekly adalimumab. She will need close monitoring and repeat imaging in the future. 

## Conclusions

Facet joint synovitis and erosion of the lumbar spine in an adolescent with no other symptoms is a unique presentation of juvenile idiopathic arthritis. This case suggests a novel differential diagnosis and emphasizes the importance of early identification and management of inflammatory back pain in adolescents. Here, we suggest control of inflammation with tumor necrosis factor (TNF) inhibitor medication could help with symptom resolution.
